# Agar and Carrageenan as Cost-Effective Gelling Agents in Yeast-Reduced Artificial Diets for Mass-Rearing Fruit Flies and Their Parasitoids

**DOI:** 10.3390/insects11020131

**Published:** 2020-02-18

**Authors:** Carlos Pascacio-Villafán, Larissa Guillén, Martín Aluja

**Affiliations:** Red de Manejo Biorracional de Plagas y Vectores, Clúster Científico y Tecnológico BioMimic®, Instituto de Ecología, A.C., Carretera Antigua a Coatepec 351, El Haya, Xalapa 91073, Veracruz, Mexico; larissa.guillen@inecol.mx (L.G.); martin.aluja@inecol.mx (M.A.)

**Keywords:** *Anastrepha ludens*, insect rearing, diet optimization, sterile insect technique, hymenopterous parasitoids

## Abstract

The development of cost-effective diets for mass-rearing fruit flies (Diptera: Tephritidae) and their parasitoids in pest control programs based on the Sterile Insect Technique is a high priority worldwide. To this end, we tested carrageenan, agar, gelatin and two types of pregelatinized corn starches as gelling agents at varying percentages in a yeast-reduced liquid larval diet for rearing the Mexfly, *Anastrepha ludens*. Only diets with 0.234% (*w*/*w*) agar or 0.424% carrageenan were identified as diets with potential for mass-rearing *A. ludens* in terms of the number of pupae recovered from the diet, pupal weight, adult emergence, flight ability and diet cost. Comparative experiments showed that yeast-reduced agar and carrageenan gel diets produced heavier pupae and higher proportions of flying adults than the standard mass-rearing diet. The gel-agar and mass-rearing diets produced more pupae than the gel-carrageenan diet, but the latter produced more suitable larvae as hosts for rearing of *Diachasmimorpha longicaudata* (Hymenoptera: Braconidae) females, a widely used fruit fly biocontrol agent. Yeast-reduced agar and carrageenan gel diets could represent cost-effective fruit fly mass-rearing diets if a practical system for gel diet preparation and dispensation at fruit fly mass-rearing facilities is developed.

## 1. Introduction

The Sterile Insect Technique (SIT) and biological control programs using hymenopterous parasitoids to control pestiferous fruit flies (Diptera: Tephritidae) worldwide, depend on a continuous supply of millions of high-quality insects that have been reared in cost-effective artificial diets [1,2]. These diets constitute the medium in which fruit fly larvae will live, feed and interact with conspecifics before metamorphosing into pupae. The pupal stage is then irradiated to produce sterile adult males that will be released in the field to compete with fertile wild males for opportunities to mate with wild female flies [1,3]. Thus, larval diets are critical for the optimal development of larvae and mass production of sexually competitive adults [1,3,4,5].

The Mexican Fruit Fly or Mexfly, *Anastrepha ludens* (Loew), is a major pest of citrus (*Citrus* spp.) and mango (*Mangifera indica* L.) [3]. *Anastrepha ludens* females lay their eggs in the pulp of most hosts (only in the case of *Casimiroa greggii* (S. Watson) F. Chiang are eggs inserted in the seeds) and, depending on the temperature, 3–12 days later, larvae hatch and start consuming the pulp [6]. Larvae go through three stages before abandoning the fruit to pupate in the ground, and adults emerge approximately 12 to 32 days later [6]. This pest is distributed from southern US throughout Mexico to Central America [6]. In Mexico, the Moscafrut facility of the National Campaign against Fruit Flies SENASICA-SADER, produces about 200 million *A. ludens* sterile flies per week for use in SIT releases [3]. In addition, millions of *A. ludens* larvae are used as hosts of the larval-pupal endoparasitoid *Diachasmimorpha longicaudata* (Ashmead) (Hymenoptera: Braconidae) which are released in augmentative biological control programs against *A*. *ludens* and *A*. *obliqua* in mango and citrus-producing areas [3,7]. Mass production of *A. ludens* at Moscafrut is currently based on a diet that includes water, sugar and yeast as nutrients, and corncob powder as a bulking agent [3,8]. The major weakness of this formula is that corncob powder is often contaminated with mycotoxins [9] as most uses of this by-product of maize do not require a high purity substrate. This has become a cause for concern because a contaminated batch of corncob powder can result in a major reduction in the rearing process as yields collapse and the stock population declines, provoking costly time lags during the recovery phase [4,9,10]. In addition, corncob powder promotes high temperatures in the diet with negative effects on larval development [10]. This highlights the need for corncob substitutes in fruit fly rearing diets as a high priority in diet development [10].

Gelling agents are another group of ingredients that are used in insect diets to transform high-water content mixtures into solids with a homogeneous distribution of materials [11]. Gelling agents can also inhibit undesirable reactions among diet ingredients [11]. Gel diets for artificial rearing of tephritids have been known for many decades [12]. In the last 13 years, several gel diet formulations have been developed for tephritid flies within the economically important genera *Anastrepha*, *Ceratitis*, *Rhagoletis* and *Bactrocera* [13,14,15,16,17,18,19]. The most common gelling agent in these diets is agar, with the drawback of its high cost [11,18]. However, the cost problem can be overcome by lowering the proportion used in the diet [14]. Gel diets containing agar and a gel diet based on a commercial mixture of pregelatinized starches have shown potential for the mass-rearing of *A. ludens* at the Moscafrut facility [16,17]. However, the substitution of the standard diet with a gel-based diet has not been implemented given its cost and the impracticalities implicit in the preparation process as agar needs to be heat-activated in boiling water and scaling-up this process requires developing new diet handling technologies [16,17]. Despite these drawbacks, the concept of gel-based diets has been integrally adopted for the mass-rearing of *Bactrocera tryoni* (Froggatt) as part of a SIT-based program in Australia [20]. The gel diet developed for this pestiferous fly yields larger numbers of adult flies, generates significantly less diet waste and reduces overall labor costs compared to a traditional diet with a bulking agent [5,19,20].

Here, we incorporated new elements into the development of gel diets for rearing of *A. ludens* by testing gelling agents such gelatin and carrageenan that show great promise due to their low cost and that were not considered in previous studies. We also examined the influence of the modified diets on the suitability of *A. ludens* larvae for rearing of the parasitoid wasp *D. longicaudata*. Given that reducing the cost of the diet is another major challenge in the Moscafrut facility [3], we followed a diet optimization perspective using a liquid diet formulation without corncob powder and reduced in yeast content to lower diet cost [8,21]. Our main goal was to determine the feasibility of developing a rearing system for *A. ludens* involving gel diets, to support the decision-making process related to the mass-rearing of this fly pest at the Moscafrut facility.

## 2. Materials and Methods

### 2.1. Experimental Flies

*Anastrepha ludens* were obtained from a laboratory colony established since 1998 at the Red de Manejo Biorracional de Plagas y Vectores of the Instituto de Ecología, A.C., Xalapa, Mexico (RMBPV). This *A. ludens* colony has been maintained on an artificial diet in the RMBPV for over 120 generations [21]. Details on the handling and maintenance of this *A. ludens* colony are described in Aluja et al. [22]. We used *A. ludens* eggs collected from artificial oviposition devices. Eggs were placed on pieces of black terylene cloth on top of moistened cotton inside an 8.5 cm diameter plastic Petri dish and incubated in a dark laboratory at 29 ± 1 °C and 70 ± 5% relative humidity for four days until they hatched, and neonate larvae were used in experiments.

### 2.2. Experimental Diets

All gel diets tested were based on a liquid diet formulation devoid of corncob powder as a bulking agent [21] and with 0.8% less yeast than the mass-rearing diet formulation to reduce costs [8]. All gel diets consisted of yeast (5.3% *w*/*w*), sugar (8.2% *w*/*w*), corn flour (6% *w*/*w*), citric acid (0.44% *w*/*w*), sodium benzoate (0.4% *w*/*w*) and methyl paraben (0.1% *w*/*w*); the remaining 79.56% (*w*/*w*) consisted of water (70.56–79.39%) plus one of the following gelling agents: pregelatinized corn starch A or B (1.6–9%) in Experiment 1; agar, carrageenan or gelatin (0.17–1.5%) in Experiments 2 and 3. The mass-rearing diet formulation used in Experiments 1–3, was based on the formulation used for mass-rearing of *A. ludens* at the Moscafrut facility [3]. This diet consisted of yeast (6.1% *w*/*w*), sugar (8.3% *w*/*w*), corn flour (5.4% *w*/*w*), corncob powder (16.3% *w*/*w*), citric acid (0.45% *w*/*w*), sodium benzoate (0.41% *w*/*w*), methyl paraben (0.1% *w*/*w*), guar gum (0.1% *w*/*w*) and water (62.84% *w*/*w*) [3]. We used 150 g portions of diet in all of the experiments. The exact formulations of the diets tested in Experiments 1–3 can be found in Appendix A, Table A1, Table A2 and Table A3, respectively. The costs and suppliers of all the ingredients used in the experimental diets are shown in Table 1.

### 2.3. Diet Preparation and Experimental Designs

#### 2.3.1. Experiment 1—Pregelatinized Corn Starch Diets

We used two types of food grade pregelatinized corn starches as gelling agents: pregelatinized starch A and pregelatinized starch B. These starches jellify at ambient temperature and differ in their viscosity which ranges between 600 to 800 centipoise (cps) in the case of pregelatinized starch A, and of 1000 to 14,000 cps in the case of pregelatinized starch B. The percentage (*w*/*w*) of either pregelatinized starch A or B in the diets ranged from 1.6 to 9% (Appendix A, Table A1). This range of pregelatinized starch percentages tested was based on preliminary tests that indicated that percentages below 1.6% and above 9%, generated diets with a liquid or a very dense consistency, respectively, from which no *A. ludens* pupae were recovered. A total of 18 different diet formulations were tested, some of which were replicated two, three or four times (Appendix A, Table A1). All dry ingredients required for 150 g of each diet were weighed individually on a digital scale (Sartorius CP64) and water was measured with a graduated cylinder and a micropipette. Except for the pregelatinized starch, all diet ingredients were mixed in a domestic blender (MagicBullet^®^) for 40 s. The resulting suspension was then mixed with the required amount of pregelatinized starch in the blender or manually if the resulting diet was too stiff to stir in the blender. Diets were dispensed in rearing trays 18 cm long by 9 cm wide by 5.5 cm high and allowed to stand for 1 h at room temperature before they were used in the experiments.

Explanatory variables were categorical (pregelatinized starch A and pregelatinized starch B) and continuous, i.e., percentage of 1.6 to 9% *w*/*w* of pregelatinized starch in the diet. The experiment was run in four blocks over time (four different days) and comprised a total of 36 experimental runs (Supplementary Material, Table S1). An experimental run consisted of a rearing tray as described before with 150 g of a specific diet (Supplementary Material, Table S1) inoculated with 0.22 mL of hatching *A. ludens* eggs in a piece of black terylene cloth. This was the volume of eggs used for rearing a colony of *A. ludens* at the RMBPV, and it is equivalent to ca. 3,098 eggs (100 μL of eggs contain an average [± SE] of 1,408 ± 44 eggs) [21]. Eggs were measured in a graduated microtube. Response variables were based on standard production and quality control parameters of artificially-reared tephritid flies used in SIT programs in many countries [3,19,23] and on diet cost: (i) cost (US$) of the gelling agents required for 1 kg of diet, (ii) time to pupation after larval separation from diet (h), (iii) number of pupae recovered, (iv) pupal weight (mg), (v) adult emergence (proportion emerged), and (vi) flight ability (proportion of fliers). In addition to its importance as internationally used production and quality parameters, these response variables were chosen because they are known to be influenced by the larval diet [1,3]. The experiment was designed with the Design-Expert^®^ 10 Software (Stat-Ease, Inc, Minneapolis, MN) and had 12 pure error degrees of freedom and 16 degrees of freedom to evaluate the lack of fit of a quadratic model capable of detecting main and interactive effects among the explanatory variables. In addition to the pregelatinized starch diets, one experimental run with the mass-rearing diet formulation was included in each block to generate baseline values for each of the response variables evaluated. This diet was prepared by hand-mixing in a plastic tray all ingredients (except water and preservatives) required for 150 g of diet until homogeneous; then, water plus preservatives diluted in the water were added to the tray and mixed with the other ingredients for approximately 3 min. Mass-rearing diets were placed in the same type of rearing trays as described before. The order of preparation of diets and the distribution of experimental runs in the different blocks are presented in Supplementary Material, Table S1.

#### 2.3.2. Experiment 2—Agar, Carrageenan and Gelatin Diets

The agar, carrageenan and gelatin used were all food grade. The percentage (*w*/*w*) of these gelling agents in the diets ranged from 0.17 to 1.5% (Appendix A, Table A2). These percentages were based on a previous study with agar gel-diets for *A. ludens* [16] and were designed to obtain a range of consistencies from soft to firm gels [19]. A total of 21 different diet formulations, each replicated between two to four times, were tested (Appendix A, Table A2). All dry ingredients except gelling agents, were hand mixed inside plastic containers until homogeneous. Each gelling agent was dissolved with the total volume of water in a beaker and heated in a microwave oven until boiling (ca. 90 s). The beaker was covered with a lid to prevent liquid escaping during boiling. After boiling, the liquid was emptied into a domestic blender (MagicBullet^®^) together with the rest of the diet ingredients and blended for 40 s. The resulting diets were dispensed in rearing trays as mentioned before and allowed to cool for 2 h at room temperature before they were used in the experiments.

Explanatory variables were categorical (agar, carrageenan and gelatin) and continuous (percentage of 0.17 to 1.5% *w*/*w* of gelling agent in the diet). The experiment was performed in five blocks over time (days) and consisted of 57 experimental runs (Supplementary Material, Table S2). Experimental runs consisted of plastic trays with diet and *A. ludens* hatching eggs as described in Experiment 1. Response variables were the same as in Experiment 1. The experiment was designed with the Design-Expert^®^ 10 Software and had 24 pure error degrees of freedom and 22 degrees of freedom to evaluate the lack of fit of a quadratic model capable of detecting main and interactive effects among the explanatory variables. Five experimental runs with the mass-rearing diet were included in blocks 3, 4 and 5, to obtain baseline values for the response variables evaluated. The order of preparation of diets and the distribution of experimental runs in the different blocks are presented in Supplementary Material, Table S2.

#### 2.3.3. Experiment 3—Gel-Carrageenan, Gel-Agar and Mass-Rearing Diets

This was a comparative experiment which only included agar and carrageenan as gelling agents at percentages of 0.234 and 0.424% (*w*/*w*), respectively (Appendix A, Table A3). These percentages were based on the results of optimization analysis from Experiment 2. The explanatory variable in this experiment was the artificial diet with three levels: (1) gel-agar, (2) gel-carrageenan and (3) mass-rearing diets (Appendix A, Table A3). The mass-rearing and gel diets were prepared as described in Experiments 1 and 2, respectively. The experiment was designed as a randomized complete block design, with 16 replicates per diet level and a total of 48 experimental runs divided in four blocks over time (days). Experimental runs were the same as described in the previous experiments. Response variables were based on *A. ludens* production and quality parameters: (i) number of pupae recovered, (ii) mean pupal weight (mg), (iii) adult emergence (proportion), and (iv) flight ability (proportion of fliers); and on *A. ludens* quality as host for the larval-pupal endoparasitoid *D. longicaudata* by measuring: (v) parasitism (proportion of parasitoids emerged) and (vi) parasitoid sex ratio (proportion of females emerged). These variables were considered in our study because it is known that female hymenopteran parasitoids have control over the sex allocation of their offspring and tend to use high-quality hosts for female production and low-quality hosts for male production [24,25,26]. The order of preparation of diets and the distribution of experimental runs in the different blocks are presented in Supplementary Material, Table S3.

### 2.4. Experimental Procedure

In Experiments 1–3, after inoculation of *A. ludens* eggs into the diets, these were incubated in a laboratory in complete darkness at 29 ± 1 °C and 70 ± 5% relative humidity (RH) for nine days. After this time, all larvae were recovered from diets by washing the contents of rearing trays with tap water through a plastic strainer. Larval separation from diet after nine days of development is a standard procedure for mass-rearing of *A. ludens* [3]. Recovered larvae were placed in a plastic rearing tray with vermiculite in a ratio of 1: 3 larvae: vermiculite. These trays were covered with pieces of pantyhose (Cannon Mills) and placed in a laboratory at 22 ± 1 °C, 70 ± 5% RH and a photoperiod of 12: 12 h L:D. In Experiments 1 and 2, pupae were recovered daily for three consecutive days, and the time to pupation was estimated as the average time at which all larvae in a given experimental run pupated within a 72-h period. In Experiment 3, pupae were recovered after 30 h (this time was selected based on results of Experiment 2). In Experiments 1–3, recovered pupae were moved to a laboratory at 26 ± 1 °C, 60 ± 5% RH, and a photoperiod of 12: 12 h L:D. When pupae reached 11 days of age, a sample of pupae (range between 1–100 pupae in Experiment 1 and 100 pupae in Experiments 2 and 3) from each diet were weighed in an analytical balance (Sartorious CP64) to estimate average pupal weight, and then used for emergence and flight ability tests [23]. For these tests, pupae were placed inside black PVC cylinders (9 cm diameter by 10 cm in height) coated inside with a thin layer of neutral talcum powder. A 6 cm diameter cardboard ring was used to contain the pupae inside cylinders. PVC cylinders with pupae were placed inside a 90 cm long by 100 cm wide by 90 cm tall mesh cage. Four sticky traps and two plastic bottle traps baited with 300 mL of grape soft drink (Sangría Casera^®^) were hung from the ceiling of the cage to capture flying flies. Adult flies emerged inside cylinders and were allowed to fly out of the tubes; individuals incapable of flying could not leave the cylinder because the talcum powder prevented flies from climbing through the cylinder. Fliers not caught in traps were removed daily manually three times a day, and the sticky traps were changed as needed. Once emergence finished, the remaining tube contents (i.e., empty puparia, dead flies and partially emerged adults) were counted.

Only in Experiment 3, a random sample of 100, eight-day-old *A. ludens* larvae, was extracted with entomological forceps from each diet to test their quality as hosts for *D. longicaudata*, the larval-pupal endoparasitoid we used to render our experimental approach more robust. After having been removed from the diets, larvae were rinsed with tap water to wash away any traces of diet and then dried with unscented paper towels. Larvae were exposed to 50, five-day-old, gravid *D. longicaudata* females without prior oviposition experience (i.e., they were naïve) inside plexiglass cages of 20 × 20 × 20 cm. Exposure to female *D*. *longicaudata* occurred in modified sandwich-type oviposition devices [22], consisting of Petri dishes lids (6 cm diameter) covered with one piece of organdy cloth (15 × 16 cm) into which *A. ludens* larvae were placed and covered with another piece of organdy cloth wrapped at the base of the Petri dish lid and held in place with a rubber band. Oviposition units were exposed to *D. longicaudata* females for 20 min. After this time, *A. ludens* larvae from each oviposition unit were placed in 125 mL plastic cups with a perforated lid and vermiculite until pupation. All the larvae that pupated (range between 40–100) were placed individually in compartmentalized plastic dishes with 100 individual cells (1.6 cm by 1.6 cm), covered with a transparent acrylic lid with perforations to allow ventilation, until fly or parasitoid adult emergence. This test was performed at 26 ± 1 °C, 60 ± 5% RH and 12:12 h L:D photoperiod. Parasitism was estimated as the proportion of parasitoids that emerged in relation to the total number of pupae placed in individual cells, and sex ratio as the proportion of females emerged in relation to the total number of emerged parasitoids.

### 2.5. Statistical Analyses

The response variables analyzed in Experiments 1, 2 and 3, are found in Supplementary Material, Table S1, Table S2 and Table S3. Block (day) was included in all analyses as a factor of no interest by itself, but that improves the sensitivity of the analyses by isolating biological variation [27].

Response Surface Methods (RSM) [28,29] were used for the analyses given the goal of diet optimization in Experiments 1 and 2. The values of each response variable were modeled as a function of the gelling agent used (categorical variable), its percentage in the diet (continuous variable) and their interactions (i.e., gelling agent × percentage). A detailed description of these analyses can be found in Pascacio-Villafán et al. [8,30]. In short, models from the linear to the sixth-order polynomial were fitted sequentially to the values of each response variable and each time a term was added, a sequential model sum of squares was used to assess the improvement in the model fit as terms were added [28]. The selected model was evaluated by analysis of variance (ANOVA) after backward elimination of non-significant terms and based on Akaike’s Information Criterion for selection of the best fitting model [31]. Model adequacy was assessed by means of diagnostic and influence plots [29]. A Box-Cox plot was used to identify if a power transformation would help to improve the behavior of model residuals [29,32]. In Experiment 1, data on the number of pupae recovered from diet were Log_10_ (y + 0.697) transformed prior to analysis, and data on adult emergence and fliers were arcsine square root transformed [32]; in this experiment, the model fitted to data on time to pupation, ignored run 6, as this run was identified as an unusual observation exerting undue influence in the estimation of model coefficients. In Experiment 2, data on adult emergence and fliers were arcsine square root transformed to improve the behavior of model residuals; in this experiment, the model fitted to data on the number of pupae recovered, ignored run 2, as this was a run from which not a single larvae was recovered on the day of larval recovery from diets, in contrast to 213 and 552 larvae recovered from other runs with the same diet formulation (Supplementary Material, Table S2).

In Experiment 2, after fitting models to the data of all response variables, we ran a multivariate optimization analysis based on a desirability function technique [28,29]. The goal of this analysis was to identify the most desirable gelling agent and its percentage in the diet that at the lowest possible cost, and in the shortest possible time, would produce more flies of higher weight and higher proportion of emergence than those produced in the mass-rearing diet. The desirability scale of this analysis ranges from zero (least desirable) to one (most desirable) [28,29]. The optimization criteria considered in this analysis are presented in Table 2. The analyses of Experiments 1 and 2 were run with the Design-Expert^®^ 10 software (Stat-Ease, Inc, Minneapolis, MN).

In Experiment 3, we fitted Generalized Linear Models (GLMs) [33] in R [34] to identify significant effects of larval diet (gel-agar, gel-carrageenan, mass-rearing) on response variables. Data on the number of pupae recovered 30 h after larval separation from diets were analyzed with a negative binomial error distribution of the MASS package [35]. Data on pupal weight (mg) were analyzed with a Gaussian error distribution after removing two atypical data points that caused severe heteroscedasticity after fit of a Gaussian GLM to the full data set. Data on adult fly emergence and fliers (proportions) were analyzed with a quasibinomial error distribution to account for overdispersion. Data on parasitism and sex ratio of *D. longicaudata* (proportions) were also analyzed with a quasipoisson error distribution to account for overdispersion. After model fit, the assumptions of normality and homoscedasticity were checked graphically by examination of model residuals. When significant effects of the predictor on the response variables were detected, we used Tukey contrasts of the multcomp package [36] to test the null hypothesis that the mean difference between levels of the predictor variable equals zero.

To gain a clearer picture of which diet formulation would be cheaper and more viable in a mass-rearing context, we used the results of Experiment 3 to estimate the number of pupae that could be recovered per kg of the gel-agar, gel-carrageenan and mass-rearing diets as: (the number of pupae recovered from 150 g of diet / 150) × 1000. The number of flying insects per kg of diet was then estimated as: (the estimated number of pupae recovered per kg of diet × proportion of emergence) × the proportion of fliers. These estimations were made for each experimental run (Supplementary Material, Table S3). The cost of 1 kg of the gel-agar, gel-carrageenan and mass-rearing diets was estimated as the sum of the relative cost of each ingredient for 1 kg of diet; the relative cost of each ingredient was the quotient of the amount in grams of that ingredient for 1 kg of diet times the cost of 1 kg of that ingredient (Table 1) by one thousand. Then, the cost of each diet required to produce one million flying insects was estimated as (1 million / the estimated mean number of flying insects per kg of diet) × the cost of 1 kg of diet. The values of the estimated number of pupae per kg of diet and of the number of flying insects per kg of diet were subjected to a one-way ANOVA with diet type (i.e., gel-agar, gel-carrageenan and mass-rearing) as the predictor variable, followed by t-test comparisons between means. These tests were performed with the Design-Expert^®^ 10 software (Stat-Ease, Inc, Minneapolis, MN).

## 3. Results

### 3.1. Experiment 1—Pregelatinized Corn Starch Diets

#### 3.1.1. Costs of Pregelatinized Corn Starch

**Figure 1:**
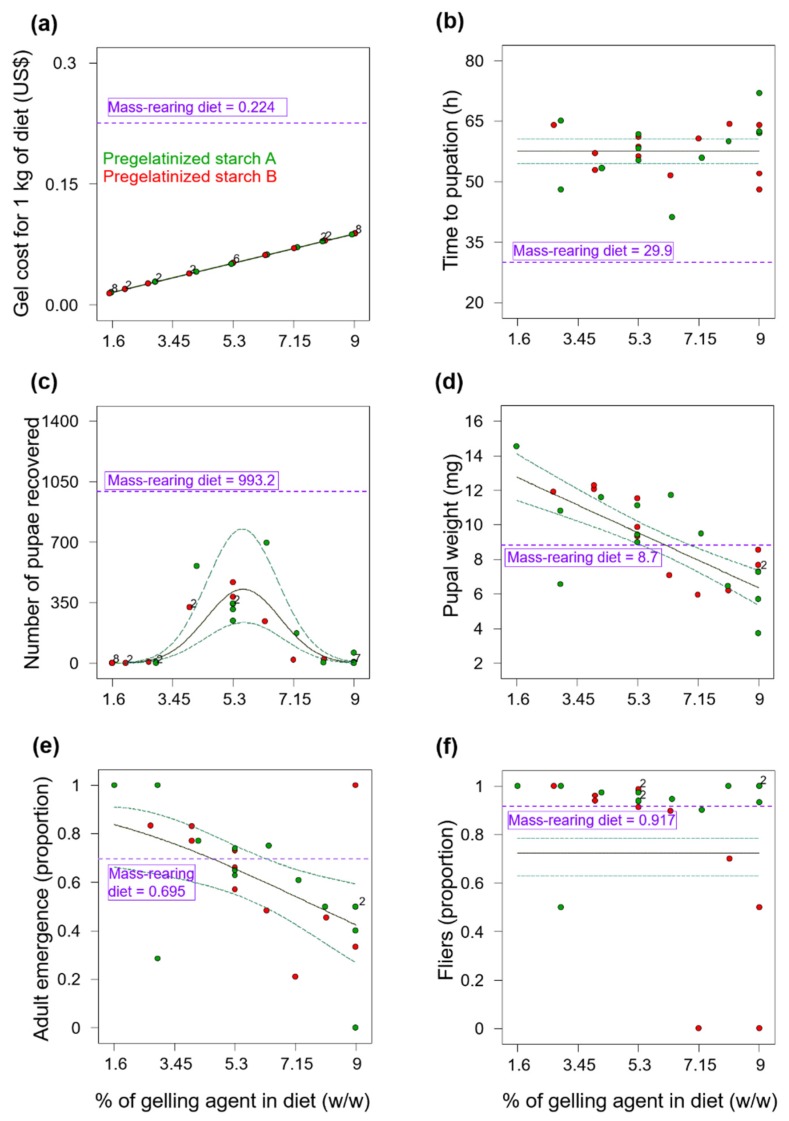
Polynomial models fitted to data on (**a**) gels costs, (**b**) time to pupation, (**c**) number of larvae recovered, (**d**) pupal weight, (**e**) adult emergence and (**f**) flight ability (fliers) of *Anastrepha ludens* as a function of the type of gelling agent (pregelatinized starch A and B) and its percentage in a yeast-reduced larval gel diet. The circles indicate the data points and the solid line the model fitted to data with 95% CI. The horizontal purple dotted line in each figure panel indicates the mean value (n = 4) observed in the mass-rearing diet, which in the case of panel (a) indicates the cost of the amount of corncob powder in 1 kg of diet.

The cost of the pregelatinized starches for 1 kg of diet ranged from US$0.016 (6% of pregelatinized starch) to US$0.090 (9% of pregelatinized starch) (Figure 1a). The pregelatinized starch ingredients used in the diets were 59.8 – 92.8% less expensive than the cost of corncob powder in the mass-rearing diet. A linear model was fitted to data on diet costs with an *R^2^* = 1.

#### 3.1.2. Time to Pupation after Larval Separation from Diet

None of the models fitted from the linear to the sixth-order polynomial pointed to significant effects of the pregelatinized starch types or their abundance in the diet on larval time to pupation (*p* ≥ 0.1998). An overall mean of 57.6 h (95% Confidence Interval [CI]: 54.6 – 60.6) provided the best description of the time to pupation in both pregelatinized starch diets and across all percentages tested (Figure 1b). On average, larvae from pregelatinized starch diets took 27.7 h longer to pupate than larvae from the mass-rearing diet.

#### 3.1.3. Number of Pupae Recovered

The number of pupae recovered from diets with the lowest and highest percentages of pregelatinized starches ranged from zero to 60, and the number of pupae recovered increased as the percentage of starch moved from the low and high extreme percentages to the central or intermediate values (Figure 1c). A reduced quadratic model (*F*_2, 30_ = 92.87, *p* < 0.0001, *R^2^_adj_* = 0.852, Figure 1c) indicated that regardless of the type of pregelatinized starch used, only the content of these starches in the diet had a significant effect on the number of pupae recovered at 72 h after larval separation from the diet (quadratic effects: *F*_1, 30_ = 169.16, *p* < 0.0001). A peak production of 428 pupae/150 g of diet was predicted by the model at a pregelatinized starch content between 5.4 and 5.7%, but we note that this yield was 57% lower than the pupal production observed with the conventional mass-rearing diet.

#### 3.1.4. Pupal Weight

A reduced linear model (*F*_1,20_ = 44.74, *p* < 0.0001, *R*^2^*_adj_* = 0.676, Figure 1d) indicated that pupal weight decreased as a function of the pregelatinized starch content in the diet. Pupal weight of individuals from diets with a content of pregelatinized starch up to 6.2% were significantly heavier than individuals from the corncob diet, whereas diets with pregelatinized starch between 6.3–9% yielded individuals with pupal weights lower than those of individuals from the mass-rearing diet (Figure 1d).

#### 3.1.5. Adult Emergence

A reduced linear model (*F*_1,20_ = 8.53, *p* = 0.0085, *R*^2^*_ad_*_j_ = 0.264, Figure 1e) indicated that adult emergence decreased as the percentage of pregelatinized starch in the diet increased; but a negative R^2^_pred_ of the model (R^2^_pred_ = -0.21) suggested that an overall mean proportion of 0.603 (95% CI: 0.493–0.705) may be a better predictor of adult emergence than the linear model fitted to the data. The linear model indicated that adult emergence of individuals from diets with a pregelatinized starch content of up to 4.5% was higher than that of individuals from the corncob diet, whereas adult emergence of individuals from diets with a pregelatinized starch content between 4.6–9% was lower than those of individuals from the mass-rearing diet (Figure 1e).

#### 3.1.6. Flight Ability

A mean model provided the best description of adult flight ability (expressed as the proportion of flies that flew out the tube, i.e., fliers) with an overall mean proportion of 0.723 (95% CI: 0.63–0.783) across all percentages and gels tested (Figure 1f). Overall, flies from the gel diets had a flight ability that was 21.1% lower than that of flies from the mass-rearing diet.

### 3.2. Experiment 2—Agar, Carrageenan and Gelatin Diets

#### 3.2.1. Costs of Gelling Agents

**Figure 2:**
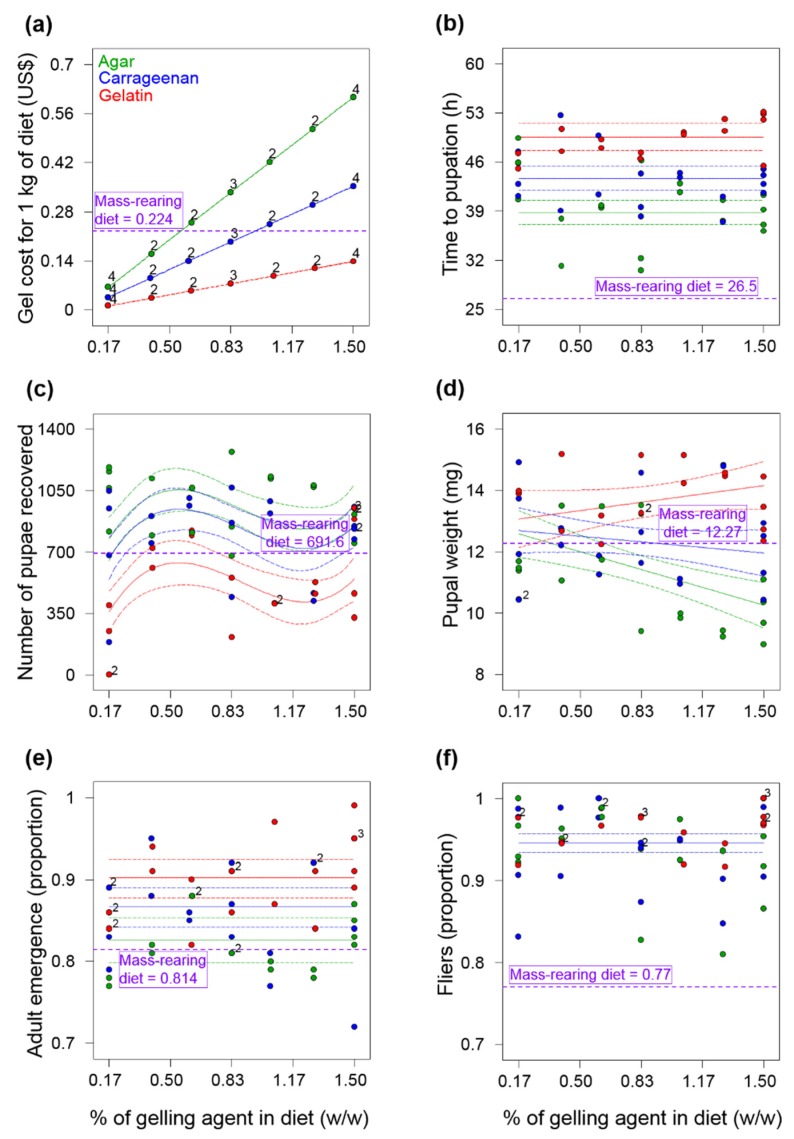
Polynomial models fitted to data on (**a**) gels costs, (**b**) time to pupation, (**c**) number of larvae recovered, (**d**) pupal weight, (**e**) adult emergence and (**f**) flight ability (fliers) of *Anastrepha ludens* as a function of the type of gelling agent (agar, carrageenan and gelatin) and its percentage in a larval gel diet. The circles indicate data points and the solid line the model fitted to data with 95% CI. The horizontal purple dotted line in each figure panel indicates the mean value (n = 5) observed in the mass-rearing diet, which in the case of panel (a) indicates the cost of the amount of corncob powder in 1 kg of diet.

The cost of the agar, carrageenan and gelatin used for 1 kg of diet ranged from US $0.069 to US $0.61, US $0.04 to US $0.36 and US $0.016 to US $0.143, respectively, across all the percentages tested (Figure 2a). Carrageenan and gelatin were 42 and 77% cheaper than agar, respectively. All percentages of gelatin used in the diet were cheaper than the percentage of corncob powder used in the mass-rearing diet, whereas in the case of agar and carrageenan only percentages below 0.55 and 0.95%, respectively, were cheaper than the corncob percentage used in the mass-rearing diet (Figure 2a). A sixth-order model was fitted to data on diet costs with an *R*^2^ = 1.

#### 3.2.2. Time to Pupation after Larval Separation from Diet

A reduced linear model (*F*_2,47_ = 33.83, *p* < 0.0001, *R*^2^*_adj_* = 0.573, Figure 2b) indicated that regardless of the gel percentage tested, larvae from the agar diets (mean = 38.79 h, 95% CI = 37.05–40.52) took significantly less time to pupate than larvae from the carrageenan (mean = 43.68 h, 95% CI = 41.99–45.37) or gelatin (mean = 49.57, 95% CI = 47.65–51.49) diets. On average, larvae from the agar, carrageenan and gelatin diets took 12.3, 17.2 and 23.1 h, respectively, longer to pupate than larvae from the mass-rearing diet (Figure 2b).

#### 3.2.3. Number of Pupae Recovered from Diets

A reduced cubic model (*F*_5,46_ = 14.94, *p* < 0.0001, *R*^2^*_adj_* = 0.577, Figure 2c) indicated significant effects of the type of gel used (*F*_2,46_ = 28.27; *p* < 0.0001) and the gel percentages tested (quadratic effects: *F*_1,46_ = 4.58, *p* = 0.0378; cubic effects: *F*_1,46_ = 7.89, *p* = 0.0073) on the number of pupae recovered at 72 h after larval separation from diets. Gelatin diets produced the lowest number of pupae across all percentages tested, whereas the agar and carrageenan diets produced the highest numbers of pupae reaching a predicted peak production of 1055 and 945 pupae/150 g of diet, respectively (Figure 2c). The peak production of the agar and carrageenan diets was 34.4 and 27%, respectively, higher than the mean pupal production from the mass-rearing diet.

#### 3.2.4. Pupal Weight

**Figure 3:**
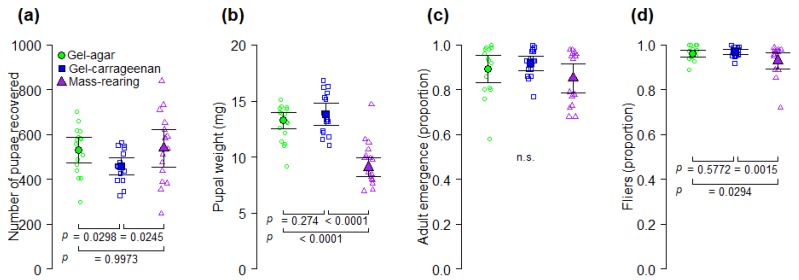
Effects of gel-agar, gel carrageenan and mass-rearing diets on (**a**) number of pupae recovered, (**b**) pupal weight (mg), (**c**) adult emergence (proportion), and (**d**) flight ability (proportion) of artificially reared *A. ludens*. Solid symbols indicate mean values and error bars show 95% CI; open symbols indicate jittered data points. The *p* values of Tukey contrasts among diets are shown within each figure panel; n.s. = no significant differences (*p* > 0.05).

A two-factor interaction model (*F*_5,44_ = 13.59, *p* < 0.0001, *R*^2^*_adj_* = 0.562, Figure 2d) indicated main (gel percentage: *F*_1, 44_ = 4.92, *p* = 0.0317; type of gel: *F*_2,44_ = 24.97, *p* < 0.0001) and interactive effects (gel percentage × type of gel: *F*_2,44_ = 6.97, *p* = 0.0023) of predictor variables on pupal weight. Pupal weight of individuals from the agar, carrageenan and gelatin diets were similar at low gel percentages but started to differ as the gel content of the diet increased (Figure 3d). Pupal weights in the gelatin diet increased as a function of the gel content, whereas pupal weight in the agar and carrageenan diets decreased as the gel percentage increased, an effect that was most apparent in the agar diet (Figure 2d).

#### 3.2.5. Adult Emergence

A reduced linear model (*F*_2,47_ = 8.13, *p* = 0.0009, *R*^2^*_adj_* = 0.225, Figure 2e) indicated that regardless of the gel percentage tested, adult emergence from the agar diets was lower than emergence from the carrageenan and gelatin diets by 4.6 and 8.3%, respectively. Adult emergence of individuals reared on agar, carrageenan and gelatin diets was greater than adult emergence of individuals reared on the corncob diet by 1.6, 6.1 and 9.7%, respectively (Figure 2e).

#### 3.2.6. Flight Ability

None of the models fitted from the linear to the sixth-order polynomial, detected significant effects of the gel type or gel percentage tested on flight ability (*p* ≥ 0.1118), and an overall mean proportion of 0.946 (95% CI: 0.933–0.957) provided the best description of flight ability across all percentages and gels tested (Figure 2f). Overall, flies from the gel diets exhibited a prevalence of flight ability that was 18% higher than that of flies from the mass-rearing diet.

#### 3.2.7. Optimization Analysis

Two results were found that meet the optimization criteria listed in Table 2: a diet with agar at a percentage of 0.234% and a diet with carrageenan at a percentage of 0.424%. The diet with agar at 0.234% had a higher desirability (*D* = 0.291) than the diet with carrageenan at 0.424% (*D* = 0.272) (Table 3).

### 3.3. Experiment 3—Gel-Agar, Gel-Carrageenan and Mass-Rearing Diets

The number of pupae recovered 30 h after larval separation from diets, was significantly affected by the type of larval diet used (deviance = 10.7; df = 2; *p* = 0.0047), with more pupae recovered from the gel-agar and mass-rearing diets than from the gel-carrageenan diet (Figure 3a). Pupal weight varied significantly as a function of the diet type used (deviance = 203.9; df = 2; *p* < 0.0001). Pupae from the gel-agar and gel-carrageenan diets were significantly heavier than pupae from the mass-rearing diet (Figure 3b). Adult emergence of flies was not significantly affected by the diet on which larvae developed (*F*_2,45_ = 2.3; *p* = 0.1084; Figure 3c). The proportion of fliers varied as a function of the diet type used (*F*_2,45_ = 5.98, *p* = 0.0051) with the gel-agar and gel-carrageenan diets producing significantly more flies capable of flying than the mass-rearing diet (Figure 3d).

The estimated number of pupae per kg of diet varied significantly as a function of the diet type (*F*_2, 42_ = 4.61, *p* = 0.0155), whereas the estimated number of flying insects per kg of diet did not differ significantly among diets (*F*_2, 42_ = 1.25, *p* = 0.2982) (Table 4). The gel-agar and gel-carrageenan diets were 24.4% and 23.7%, respectively, less expensive than the conventional mass-rearing diet (Table 4). The cost of the mass-rearing diet required to produce one million flying insects was US $66.9 and US $42 more expensive than the gel-agar and the gel carrageenan diets, respectively (Table 4).

**Figure 4:**
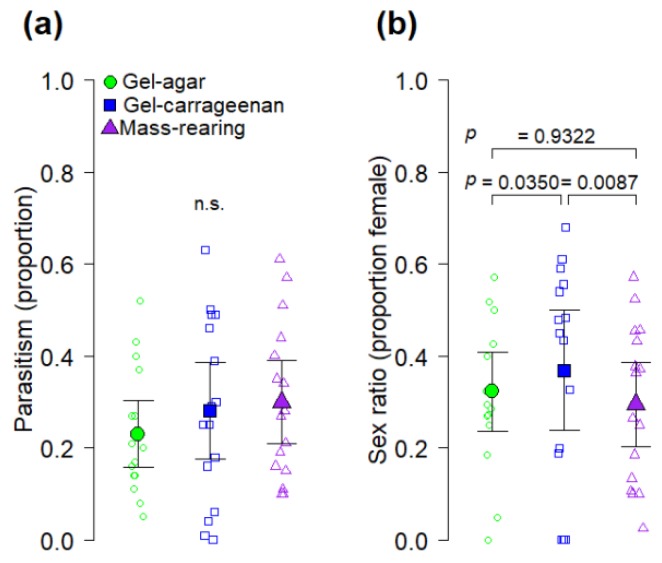
Effects of host *A. ludens* larval diet (gel-agar, gel-carrageenan and mass-rearing) on (**a**) parasitism and (**b**) sex ratio of *Diachasmimorpha longicaudata*. Solid symbols indicate mean values and error bars show 95% CI; open symbols indicate jittered data points. The *p* values of Tukey contrasts among diets are shown within each figure panel; n.s. = no significant differences (*p* > 0.05).

Finally, parasitism by *D. longicaudata* was not significantly affected by the type of diet used to rear its larval host (*F*_2,45_ = 0.71, *p* = 0.4974, Figure 4a). Importantly, the sex ratio of emerged parasitoids varied significantly as a function of the larval host diet (*F*_2,44_ = 4.5, *p* = 0.0164) with a higher proportion of females emerging from hosts reared on the gel-carrageenan diet than from hosts reared on the gel-agar and mass-rearing diets (Figure 4b).

## 4. Discussion

Although the use of gel diets for rearing the fruit fly pest *A. ludens* had been previously reported [16,17], this is the first study in which several gelling agents (carrageenan, agar, gelatin and pregelatinized corn starch) have been tested from a multivariate optimization perspective, considering production and quality parameters of both flies and parasitoids, as well as diet costs. In addition, this is the first report on the use of carrageenan and gelatin as gelling agents in an *A. ludens* larval gel diet. Our results indicate that yeast-reduced gel diet formulations based on carrageenan and agar as gelling agents can be used for more cost-effective rearing of *A. ludens* (Figure 2 and Figure 3). Even though agar and carrageenan are relatively expensive [11], the low percentages of these ingredients and the reduction in the yeast content in the diets we tested in Experiment 3 (Appendix A, Table A3), made our gel diets 24% less expensive than the mass-rearing diet. More importantly, our analyses indicate that the production of one million flying insects in the gel-agar and gel-carrageenan diets could be 27% and 17%, respectively, less expensive than with the mass-rearing formulation (Table 4). By reducing the cost of the diet, we addressed one of the most pressing priorities in mass-rearing facilities of fruit flies worldwide such as the Moscafrut facility [1,3,37]. On the other hand, although gelatin and pregelatinized corn starches were quite inexpensive, gel diets based on these ingredients did not produce sufficient numbers of flies (Figure 1c and Figure 2c) to be considered as candidate diets for large scale production of *A. ludens*.

Our results contrast with those of Rivera et al. [17], who found that a pregelatinized starch diet based on a commercial product (Nutrifly^®^) increased yields of *A. ludens* compared to a standard mass-rearing diet with corncob fractions. The exact content of yeast and the composition of pregelatinized starches in this pregelatinized starch diet were not stated [17], but it is possible that it contained starches from various sources [38]. Mixing corn starch with other types of starches or gelling agents in the diet might provide positive results for the rearing of *A. ludens* but increasing the number of ingredients would rise the cost of the diet and add unwanted complexity to the diet preparation process [10].

Overall, larvae from the gel diets in Experiments 1 and 2, took much longer to pupate than larvae from the mass-rearing diets (Figure 1b and Figure 2b). This delayed larval development time could be associated with the reduction of yeast (i.e., the source of protein) content in gel diets compared to the yeast content in the mass-rearing diet (Appendix A, Table A1 and Table A2). In fact, the duration of tephritid larval development is highly dependent on the quantity and quality of protein in the larval diet [39]. However, if the reduction of yeast content in the gel diets would have caused a nutritional deficiency in the larvae, then individuals from the mass-rearing diet with a higher level of yeast would have had higher pupal weights than individuals reared on the gel diets, but that was not what we observed. Pupal weight of flies reared on gel diets was significantly higher than that of flies reared on the mass-rearing diet (Figure 3b). Alternatively, larvae from the mass-rearing diet may have pupated earlier due to an increase in the diet temperature promoted by corncob powder as the larvae grew [10]. In any case, we note that longer larval development times could delay production times at the mass-rearing facility level, but this could be overcome by increasing the rearing temperature [8].

Despite the delay in the time to pupation of flies reared on the gelatin diets in Experiment 2 (Figure 2b) and the low numbers of pupae recovered from these diets (Figure 2c), we note that gelatin diets produced the heaviest *A. ludens* pupae and that the pupal weight increased as the percentage of gelatin in the diet increased (Figure 2d). Thus, although gelatin apparently has no essential amino acids for insects [11], it is a protein with positive effects on pupal weight of *A. ludens*. Overall, pupal weights were atypically low in our study, likely because the volume of eggs we inoculated into the experimental diets was rather high. Indeed, it was previously reported that pupal weight of *A. ludens* was reduced when flies were reared at a density of 15 larvae per g of diet compared to flies reared at a density of 10 larvae per g of diet [17], and the volume of eggs we inoculated to all the experimental diets was equivalent to approximately 14 larvae per g of diet. This high larval density inoculated to the diets and the overall positive effects of agar and carrageenan gel diets on *A. ludens* production and quality compared to the mass rearing diet (Figure 2 and Figure 3), suggest that agar and carrageenan increase the production capacity of *A. ludens* larval diet. Further studies are warranted to examine the effects of larval density in gel-agar and gel-carrageenan diets on survival and quality of *A. ludens* and to determine the optimal larval density at which the production and quality (e.g., pupal weight) of flies is maximized.

Thanks to our modeling procedure, the percentage of agar (0.234%) and carrageenan (0.434%) used in Experiment 3 was lower than the percentages of agar used in many other tephritid larval gel diets, greatly reducing the cost of this expensive ingredient. For example, gel diets for *B. tryoni* [5], *A. fraterculus* [18] and previous gel diets for *A. ludens* [16], contained 0.77%, 0.17% and 0.77–2.27% more agar, respectively, than the agar content of 0.234% we used in Experiment 3. This highlights the benefit of using RSM [28,29] to design diet experiments and, in general, to optimize insect diet formulations. These methods allow for the design of experiments involving many variables, optimizing the critical ones simultaneously [28]. Here for example, we were able to test 21 different gel diets in a single experiment (Experiment 2, Figure 2, Appendix A, Table A2) and identified optimal formulations reaching multiple optimization criteria simultaneously.

**Figure A1:**
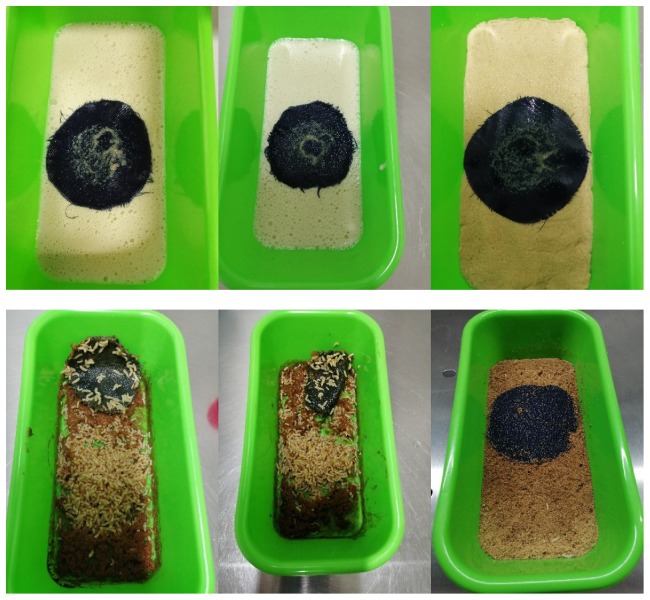
From left to right: gel-agar, gel-carrageenan and mass-rearing diets tested in Experiment 3. The top row shows the diets on the day of their preparation and inoculation with *A. ludens* hatching eggs; the bottom row shows the same diets after nine days. Note the relatively high amount of diet waste in the mass-rearing diet compared to the gel diets.

We believe that gel diets better simulate the natural conditions experienced by fruit fly larvae, as pulp is a moist medium with a smooth consistency that allows larvae to move with relative ease and ingest food. In fact, we noticed that larvae reared on the gel-agar and gel-carrageenan diets we tested, leave very little or no residues as they eat almost all the food provided (Appendix A, Figure A1), as was previously reported for *B. tryoni* reared on a gel diet with agar [5]. This contrasts with the large amounts of residues observed in diets prepared with corncob powder as a bulking agent which can comprise some 32% (*w*/*w*) of the starting amount of diet (C. Pascacio-Villafán, personal observation). This represents not only a waste of food resources and money, but also a source of pollution as the trays with diet waste are washed with water that flows into water treatment facilities or sometimes directly into the sewage system. Thus, the gel diets reported here could contribute to reduce pollution in mass-rearing facilities.

Perhaps the reason why *A. ludens* larvae eat almost all the food in the gel diets and waste so much in the mass-rearing diet (Appendix A, Figure A1) is because the mass-rearing diet using corncob powder as a bulking agent make swallowing more difficult for larvae, thus reducing the feeding rate. As a result, larvae reach the third instar having ingested less food, a fact that does not necessarily cause a drop in weight due to the high protein and carbohydrate content of artificial diets. Another possibility is that gel diets facilitate larval movement and reduce metabolic heat allowing larvae to group and via a social facilitation phenomenon increase per capita ingestion which could explain why almost no residues are left. These ideas merit further investigation.

Another novel aspect of our study is represented by the fact that, in addition of having addressed the fly perspective, we also examined the quality of *A. ludens* larvae reared on gel diets as hosts of the parasitoid wasp *D. longicaudata*. This parasitoid species is the most widely used biocontrol agent against tephritid pests worldwide [40,41]. We found that the gel-carrageenan diet produced *A. ludens* hosts from which a significantly higher proportion of *D. longicaudata* females emerged compared to the proportion of females emerging from hosts reared on the gel-agar and the mass-rearing diets (Figure 4b). The reason why *A. ludens* from the gel-carrageenan diet produced higher proportions of female wasps than the other diets is not clear in our study, but results suggest that larvae reared on this diet were of higher quality as hosts for *D. longicaudata.* Perhaps the gel-carrageenan diet improved nutrient assimilation by *A. ludens* larvae making them more nutritionally adequate for the development of female parasitoids. In fact, an in vitro study showed that some types of carrageenan modulate the digestion of macronutrients such as lipids [42]. If true for *A. ludens*, future studies should find that *A. ludens* larvae fed on the gel-carrageenan diet have a higher nutrient content than larvae from the gel-agar or mass-rearing diets. Another relevant point is that the heaviest flies in our study were obtained from the gel-carrageenan diet from which the highest proportion of female parasitoids were also obtained (Figure 3b and Figure 4b) However, we note that sex ratio of artificially reared *D. longicaudata* is apparently not affected by host size [43,44], but larger *A. ludens* hosts are known to produce female-biased sex ratios under superparasitism conditions [44]. Several studies have addressed the role of host quality (i.e., fruit fly larvae) on the fitness and performance of adult parasitoids, and how in turn the rearing media used to produce the fly larvae determines to a large extend their quality [45,46,47,48]. Future studies on the effects of the artificial larval diet of tephritid flies on hymenopteran parasitoids used in integrated pest management programs, should consider the examination of other quality parameters of parasitoids such as fecundity and longevity in the absence of food [43].

Our research provides novel gel diet formulations devoid of the troublesome corncob powder bulking agent and reduced in yeast content that could contribute to improving the cost-effectiveness of the SIT and biological control programs against *A. ludens* in Mexico. Gel diets developed in this study could also be used as cost-effective generic diets for rearing other pestiferous *Anastrepha* spp. [10,49]. Having cost-effective diets for mass-rearing fruit flies becomes particularly relevant under current climate change conditions that is expanding the distribution and host range of pestiferous flies [6,50], as this may require increasing the mass production of artificially-reared sterile flies and parasitoids to protect fruit growing areas that were not previously attacked.

## 5. Conclusions

We conclude that yeast-reduced gel diets with 0.234 % agar or 0.424 % carrageenan (Appendix A, Table A3) are significantly less expensive and can produce significantly more and better-quality flies than the *A. ludens* mass-rearing diet formulation currently used at Moscafrut. The gel-agar diet formulation (Appendix A, Table A3) was the most economical and viable for rearing large numbers of flying insects (Table 4). Gel diets could provide a better medium for optimal larval development than diets with bulking agents used worldwide for mass-rearing fruit flies which in turn provides the important side benefit of generating high-quality hosts for mass-rearing parasitoids. Additional research is required to develop a practical and cost-effective system for gel diet preparation and handling at Moscafrut, as the current procedure based on a diet with corncob powder as bulking agent would not be compatible with the new technology. If a gel diet system could be implemented for mass-rearing of *A. ludens*, we can envision further improvements in the production process of flies used in SIT releases and of parasitoids used in biocontrol programs. Moving from a mass-rearing system with a corncob diet to a system with a gel diet represents an important challenge. However, the Moscafrut facility has been an example of continuous improvements and development of new methodologies [3], so we have no doubt that the challenge could be easily overcome. Our approach could also be applied in other fruit mass-rearing facilities worldwide, as the principles applied in our study, including our modeling procedure, are of general application.

## Figures and Tables

**Table 1:** Costs per kg and suppliers of the ingredients used in experimental diets.

Ingredient	Cost Per kg (US $) ^1^
Torula yeast ^2^	6.03
Sugar ^3^	1.00
Corn flour ^4^	0.52
Corncob powder ^5^	1.4
Citric acid ^6^	1.65
Sodium benzoate ^7^	3.1
Methyl paraben ^8^	10.8
Guar gum ^9^	5.3
Pregelatinized corn starches A and B ^10^	1.005
Agar ^11^	40.67
Carrageenan ^12^	23.76
Gelatin ^13^	9.52

^1^ Mexican peso/US dollar exchange rate at the time of manuscript preparation was US$1 = $18.91 Mexican pesos. ^2^ Lallemand, Mexico, S.A. de C.V. ^3^ Ingenio Huixtla, S.A., Chiapas, Mexico. ^4^ Minsa, S.A. de C.V., Arriaga, Chiapas, Mexico. ^5^ Coltec Inter S. de R.L. de C.V., Guadalajara, Jalisco, Mexico. ^6^ Asesoría y Abastecimiento de México, S.A. de C.V. ^7,8^ Industria Química del Centro, S.A. de C.V., Mexico. ^9^ Cava Nutrielementos, S.A. de C.V., Mexico. ^10^ Adisa, Almidones y Desarrollos Industriales Estado de México, Mexico. ^11,12^ Quial, Aditivos Alimentarios, Tepic, Nayarit, Mexico. ^13^ La Serpentina, Veracruz, Mexico.

**Table 2:** Optimization criteria used to find an optimal gel diet formulation for rearing of *Anastrepha ludens*.

	Optimization Criteria			
Response Variable	Goal	Lower Limit	Upper Limit	Importance
Number of pupae recovered	Maximize	692	1270	5
Pupal weight (mg)	Maximize	12.27	15.17	4
Time to pupation (h)	Minimize	30.5	45	3
Adult emergence (proportion)	Maximize	0.72	0.97	3
Gel cost (US $)	Minimize	0.00243	0.09151	3
Fliers (proportion) ^1^	None	-	-	-

^1^ The model fitted to these data indicated no significant difference among gelling agents and across proportions tested, thus this response variable was not considered for optimization purposes.

**Table 3:** Desirability values of the optimization analysis for the results of agar at 0.234% and carrageenan at 0.424%.

Response Variable	Desirability ^1^	
	Agar 0.234%	Carrageenan 0.424%
Pupae recovered (#)	0.309	0.395
Pupal weight (mg)	0.07	0.097
Time to pupation (h)	0.429	0.091
Adult emergence (proportion)	0.395	0.544
Gel cost (US $)	0.869	0.857
Combined	0.291	0.272

^1^ Ranges from 0 (undesirable) to 1 (very desirable) and its value explain how closely the lower and upper limits of the optimization criteria are set relative to the actual optimum.

**Table 4:** Mean values (± SE) for the estimated number of pupae and the number of flying insects per kg of diet, and the costs of 1 kg of diet and of the diet required to produce 1 million of flying insects.

Diet	Estimated Number of Pupae Per kg of Diet ^1^	Estimated Number of Flying Insects Per kg of Diet ^1^	Cost of 1 kg of Diet (US $)	Cost of the Diet Required to Produce 1 Million of Flying Insects (US $)
Mass-rearing	3593.3 (±259.7) a	2982.6 (±321.6) a	0.74	248.0
Gel-agar	3544.6 (±177.8) a	3085.8 (±224.5) a	0.559	181.1
Gel-carrageenan	3065.8 (± 119.9) b	2741.1 (± 127.9) a	0.564	205.9

^1^ Means labeled with identical letters within each column indicate no significant differences for one-way ANOVA (t-test contrasts, *p* > 0.05).

## Supplementary Materials

The following are available online at https://www.mdpi.com/2075-4450/11/2/131/s1, Table S1: response variables analyzed in Experiment 1; Table S2: response variables analyzed in Experiment 2; Table S3: response variables analyzed in Experiment 3.

## Appendix A

**Table A1:** Gel diet formulations tested in Experiment 1. The values of diet ingredients are given as percentages (*w*/*w*).

Diet No.^1^	Water	Gelling Agent	Type of Gelling Agent	No. of Experimental Runs
1	77.96	1.60	Pregelatinized starch A	4
2	77.54	2.02	Pregelatinized starch A	1
3	76.84	2.72	Pregelatinized starch A	1
4	75.59	3.97	Pregelatinized starch A	2
5	74.26	5.30	Pregelatinized starch A	3
6	73.28	6.28	Pregelatinized starch A	1
7	72.41	7.15	Pregelatinized starch A	1
8	71.47	8.09	Pregelatinized starch A	1
9	70.56	9.00	Pregelatinized starch A	4
10	77.96	1.60	Pregelatinized starch B	4
11	77.54	2.02	Pregelatinized starch B	1
12	76.62	2.94	Pregelatinized starch B	2
13	75.38	4.18	Pregelatinized starch B	1
14	74.26	5.30	Pregelatinized starch B	3
15	73.23	6.33	Pregelatinized starch B	1
16	72.31	7.25	Pregelatinized starch B	1
17	71.49	8.07	Pregelatinized starch B	1
18	70.56	9.00	Pregelatinized starch B	4

^1^ All diets had constant percentages (*w*/*w*) of yeast (5.3%), sugar (5.2%), corn flour (6%), sodium benzoate (0.4%), methyl paraben (0.1%) and citric acid (0.44%). All ingredients sum to 100% of the total diet.

**Table A2:** Gel diet formulations tested in Experiment 2. The values of diet ingredients are given as percentages (*w*/*w*).

Diet No. ^1^	Water	Gelling Agent	Type of Gelling Agent	No. of Experimental Runs
1	79.39	0.17	Agar	4
2	79.16	0.40	Agar	2
3	78.94	0.62	Agar	2
4	78.73	0.84	Agar	3
5	78.51	1.05	Agar	2
6	78.28	1.28	Agar	2
7	78.06	1.50	Agar	4
8	79.39	0.17	Carrageenan	4
9	79.16	0.40	Carrageenan	2
10	78.95	0.61	Carrageenan	2
11	78.73	0.84	Carrageenan	3
12	78.51	1.05	Carrageenan	2
13	78.28	1.28	Carrageenan	2
14	78.06	1.50	Carrageenan	4
15	79.39	0.17	Gelatin	4
16	79.16	0.40	Gelatin	2
17	78.94	0.62	Gelatin	2
18	78.73	0.84	Gelatin	3
19	78.49	1.07	Gelatin	2
29	78.27	1.29	Gelatin	2
21	78.06	1.50	Gelatin	4

^1^ All diets had constant percentages (*w*/*w*) of yeast (5.3%), sugar (5.2%), corn flour (6%), sodium benzoate (0.4%), methyl paraben (0.1%) and citric acid (0.44%). All ingredients sum to 100% of the total diet.

**Table A3:** Diet formulations tested in Experiment 3; each diet treatment was replicated 16 times. The values of diet ingredients are given as percentages (*w*/*w*).

Diet Type	Yeast	Sugar	Corn Flour	Sodium Benzoate	Methyl paraben	Citric Acid	Water	Agar	Carrageenan	Corncob Powder	Guar Gum
Gel-Agar	5.3	8.2	6	0.4	0.1	0.44	79.326	0.234	-	-	-
Gel-Carrageenan	5.3	8.2	6	0.4	0.1	0.44	79.136	-	0.424	-	-
Mass-rearing	6.1	8.3	5.4	0.41	0.1	0.45	62.84	-	-	16.3	0.1
